# Shedding Light on the COVID-19 Pandemic: Placental Expression of Cell Biomarkers in Negative, Vaccinated, and Positive Pregnant Women

**DOI:** 10.3390/jcm13185546

**Published:** 2024-09-19

**Authors:** Constantin Condac, Ludmila Lozneanu, Daniela Roxana Matasariu, Alexandra Ursache, Iuliana Elena Bujor, Maria Elena Niță, Vasile Lucian Boiculese, Victoria Bîrluțiu

**Affiliations:** 1 Department of Anesthesia and Intensive Care, “Cuza Vodă” Hospital, 700038 Iasi, Romania; costicondac@gmail.com; 2 Department of Infectious Diseases, University of Medicine and Pharmacy “Lucian Blaga”, 550169 Sibiu, Romania; victoria.birlutiu@ulbsibiu.ro; 3 Department of Morpho-Functional Sciences I—Histology, University of Medicine and Pharmacy “Gr. T. Popa”, 700115 Iasi, Romania; ludmila.lozneanu@umfiasi.ro; 4 Department of Obstetrics and Gynecology, University of Medicine and Pharmacy “Gr. T. Popa”, 700115 Iasi, Romania; iuliana-elena.bujor@d.umfiasi.ro; 5 Department of Obstetrics and Gynecology, “Cuza Vodă” Hospital, 700038 Iasi, Romania; elemarni28@gmail.com; 6 Biostatistics, Department of Preventive Medicine and Interdisciplinarity, University of Medicine and Pharmacy “Gr. T. Popa”, 700115 Iasi, Romania; lboiculese@gmail.com

**Keywords:** COVID-19, placenta, immunohistochemistry, diagnosis, SARS-CoV-2, Pfizer vaccine, CD44, osteopontin, COX2

## Abstract

**Background**: We investigated the expression of inflammation, placental development, and function markers, including cluster of differentiation 44 (CD44), osteopontin (OPN), and cyclooxygenase-2 (COX-2), to shed light on the controversy regarding the impact of the COVID-19 epidemic on fetal development and pregnancy outcomes. **Methods**: We immunohistochemically analyzed placental tissue from 170 patients (65 COVID-positive and unvaccinated women; 35 Pfeizer-vaccinated and COVID-negative women; and 70 COVID-negative and unvaccinated women, without any other associated pathology) for particularities in the expression of these three molecules. **Results**: CD44 expression was highest in COVID-negative and unvaccinated women, moderate in COVID-positive cases, and lowest in vaccinated and COVID-negative women. OPN expression was highest in COVID-negative and Pfeizer-vaccinated cases, moderate in COVID-negative and unvaccinated cases, and lowest in COVID-positive cases. COX-2 expression was increased in COVID-negative and unvaccinated women, modestly elevated in COVID-positive and unvaccinated cases, and lowest in vaccinated cases. **Conclusions**: These findings reflected an alteration in the placental structure and consequent function due to altered expression of the three studied molecules.

## 1. Introduction

The impact of coronavirus disease (COVID) infection on pregnant women and their infants is of particular interest to obstetricians, pediatricians, and patients [1]. In past epidemics of emerging infections, the pathological analysis of placentas has proven to be a highly informative technique for understanding the mechanism of transmission to the fetus [2].

This infection manifests with a wide range of organ damage, resulting in a diverse constellation of clinical conditions [3]. Human cell viral access is mediated by angiotensin-converting enzyme 2 (ACE-2), receptors for which are abundant on the placental syncytiotrophoblast, underlying a potential connection between the infection and altered placental function [4,5]. Although the pathophysiology is not yet completely understood, there is evidence that the timeframe in which infection appears might play a crucial role in maternal and fetal outcomes. Less mature placentas seem to be more vulnerable to certain types of viral-mediated damage than mature placentas, due to the systemic inflammatory response and hypercoagulation [6].

Cluster of differentiation 44 (CD44) is a widespread, well-known molecule that plays a key role in many cell processes (regulation of vascular permeability, inflammatory response, signaling pathways, cell activation, cell adhesion, cell migration, and cell-to-cell and cell-to-matrix interaction). Hyaluronic acid (HA) is the intermediary for cell-to-cell and cell-to-matrix CD44 interactions [7,8,9]. This molecule can exhibit other ligands as well, and is capable of interactions with osteopontin, collagen, and matrix metalloproteinases. The CD44 molecule seems to be involved in the stabilization and orientation of the HA grid, both of which are essential in maintaining the placental integrity in normal pregnancy [8,9].

Osteopontin (OPN) is another important CD44 counterpart. It is a major non-collagenous bone matrix protein that is involved in both normal and pathological calcification processes, and also in angiogenesis via CD44 and integrin interactions [10,11]. In the placenta tissue, this effector molecule is expressed by the cytotrophoblasts of chorionic villi, augmenting both ion and nutrient transport [12]. Although osteopontin expression is only present in immature tissue, it becomes visible in a series of processes such as wound healing and pathological calcification (atherosclerosis, breast cancer, and renal stones) [10].

As a part of the cyclooxygenase (COX) family, cyclooxygenase-2 (COX-2) is the base enzyme responsible for the synthesis of prostaglandins from arachidonic acid. Its involvement in angiogenesis, by promoting the proliferation of vascular endothelial cells, inhibiting inflammation, and cell apoptosis, has been acknowledged. It has been demonstrated that abnormal COX-2 levels are associated with ovulation failure, infertility, and implantation disorders [13]. It is an important marker for decidualization, and is highly expressed around the embryo invasion site [14].

The study of CD44, OPN, and COX-2 expression in placental tissue might provide valuable insights into the effects of COVID infection and vaccination during pregnancy. Each marker plays a distinct role in cellular processes and immune responses, making them crucial for understanding placental pathology under different conditions. Our goal was to evaluate CD44, OPN, and COX-2 expression in three groups of pregnant women (healthy and unvaccinated, COVID-positive and unvaccinated, and COVID-vaccinated and COVID-negative) to assess possible physiopathological pathways for the alterations in placental architecture consecutive to inflammatory responses, which may be influenced by viral infections or vaccine-induced immune modulation.

## 2. Materials and Methods

### 2.1. Patients and Tissue Samples

The study period was from January 2021 to January 2023. All of the patients provided written informed consent. The study was by the Ethics Committee of the University of Medicine and Pharmacy “Lucian Blaga”, Sibiu (1442/19.03.2024) and of the Obstetrics and Gynecology Hospital “Cuza-Voda” in Iasi, Romania (10426/24.08.2021 and 19/04.08.2023). 

Specimens were collected from women who gave birth at term without complications, associated disease, or chronic treatment (toxoplasma, rubella, cytomegalovirus, herpes with no acute infection detected; hepatitis B and C, HIV, and syphilis-negative women). Formalin-fixed, paraffin-embedded tissue samples were collected from pregnant women who gave birth at the Obstetrics and Gynecology Hospital “Cuza-Voda” in Iasi.

The pregnant women were divided into three groups: COVID-positive and unvaccinated women, COVID-negative and vaccinated women, and COVID-negative and unvaccinated women. The COVID-positive and unvaccinated group included asymptomatic women, with the disease detected anytime during pregnancy but at least 14 days before birth. The diagnosis of COVID positivity was established using Polymerase Chain Reaction (PCR) testing. The second group included Pfeizer-vaccinated women, with the first or second dose administered at least 14 days before birth, who never tested positive for COVID during the pandemic, and with a negative rapid COVID test confirmed every three weeks during pregnancy. The control group included COVID-negative and unvaccinated women who gave birth at term without any complications or associated pathology (all the women included in the third group had not tested positive during the pandemic until inclusion in our study, and had negative rapid COVID tests every three weeks during pregnancy).

#### Exclusion Criteria

Women with obstetrical and/or other medical complications were excluded from our analysis. Patients with COVID infection at the time of giving birth; malignancy; depression; genetic syndromes; infectious or autoimmune diseases; pre-existing or gestational diabetes; hypertension and its complications, such as preeclampsia; premature rupture of membranes; oligohydramnios; intrauterine growth restriction, defined as ultrasound estimated fetal weight less than the 10th percentile for gestational age; chorioamnionitis; and smoking were excluded.

### 2.2. Immunohistochemistry

We collected four tissue samples from each placenta, one for each of the four quadrants. Hematoxylin and eosin (H&E) sections were examined, and immunohistochemistry (IHC) was evaluated by two pathologists. IHC staining was performed on formalin-fixed, paraffin-embedded tissues for immunohistochemistry utilizing monoclonal antibodies against cluster of differentiation 44 (CD44), osteopontin (OPN), and cyclooxygenase-2 (COX-2). Four-micrometer-thick serial sections were prepared in citrate buffer (pH 6) after deparaffinization in xylene and rehydration in ethanol series. Endogenous peroxidase activity was inhibited with 0.3% H_2_O_2_ for 20 min at room temperature. IHC was used to determine the expressions of CD44, OPN, and anti-COX2 using specific Abcam Company dilutions for CD44 of 1:250 (catalog no. ab157107, Abcam, Cambridge, UK), OPN of 1:200 (catalog no. ab8448, Abcam, Cambridge, UK), and anti-COX-2 of 1:100 (catalog no. ab15191, Abcam, Cambridge, UK), and were incubated overnight at 4 °C. The sections were washed, exposed to the secondary antibody for 45 min at 37 °C, and cleaned with phosphate-buffered saline (PBS). Hematoxylin was used as a counterstain in the standard avidin–biotin–peroxidase technique, using a liquid DAB (diaminobenzidine) substrate and chromogen system. Human jejunum served as a positive control for CD44, OPN, and anti-COX-2.

All the placental samples were examined for CD44, OPN, and anti-COX-2 presence. Positive cells (brown or yellowish-brown color in the nucleus) in the epithelial and stromal compartments were considered CD44, OPN, and anti-COX-2 positive, regardless of staining intensity or the number of positive cells.

### 2.3. Statistical Analysis

The data were imported into Microsoft Excel and analyzed in SPSS 24 (IBM Corp. Released 2016. IBM SPSS Statistics for Windows, Version 24.0. IBM Corp., Armonk, NY, USA). The descriptive statistics included sample size (N absolute and N% relative frequencies), mean, standard deviation, and the 95% confidence interval for the mean, quartiles, minimum, and maximum. Statistical hypothesis tests included nonparametric tests like Kruskal–Wallis for three-sample and post hoc tests for two-sample comparisons with Bonferroni–Dunn corrections. These were applied to the analysis of continuous numerical variables. The Chi-square or Fisher exact tests were used for categorical variables. Pearson’s correlation coefficient was used to evaluate the correlation between biomarkers. A standard significance level of 0.05 was used.

## 3. Results

**Figure 1:**
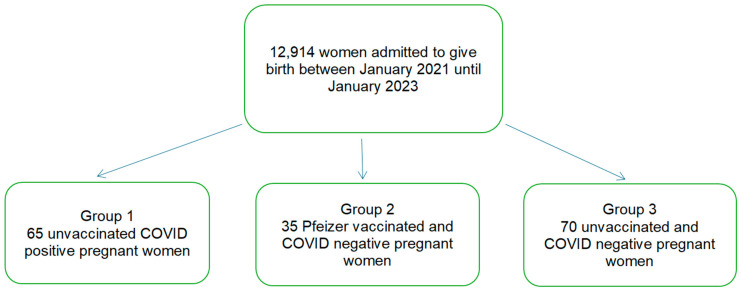
Flow chart of our included cases.

After applying the inclusion and the exclusion criteria, 170 pregnant women were included in the study: 65 unvaccinated COVID-positive women in group 1, 35 vaccinated and COVID-negative women in group 2, and 70 unvaccinated COVID-negative women in group 3. All of the COVID-positive study patients were asymptomatic. The ages of our patients ranged from 18 to 39 years (Figure 1).

All of the study patients were asymptomatic. None required supplemental oxygen administration or admission to the intensive care unit. Laboratory testing, such as complete blood count, inflammatory markers, and biochemical profile, was performed. Neonatal outcomes, including birthweight, APGAR scores, and neonatal intensive care unit (NICU) admissions, were similar between the three groups (Table 1).

**Figure 2:**
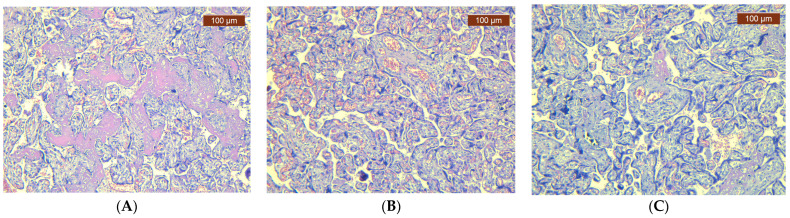
Representative histopathological changes in the placenta (HE). (**A**) COVID-19-positive pregnant women: small, well-vascularized chorionic villi. Syncytial knots and intervillous fibrin (HEx10). (**B**) COVID-19-vaccinated pregnant women: chorionic villi, congestion, and fibrosis (HEx20). (**C**) COVID-19-negative and unvaccinated pregnant women: different size of chorionic villi, congestion, and area of fibrosis (HEx10).

When examining the H&E sections, pathological changes were noted. In COVID-positive and vaccinated women, we observed signs of inflammation, such as villitis or intervillositis, increased fibrin deposition, and possibly microthrombi. These changes indicated an immune response or vascular involvement associated with COVID. Conversely, in non-COVID and unvaccinated women, the placenta showed typical histological features without these specific inflammatory or vascular alterations (Figure 2A–C).

**Figure 3:**
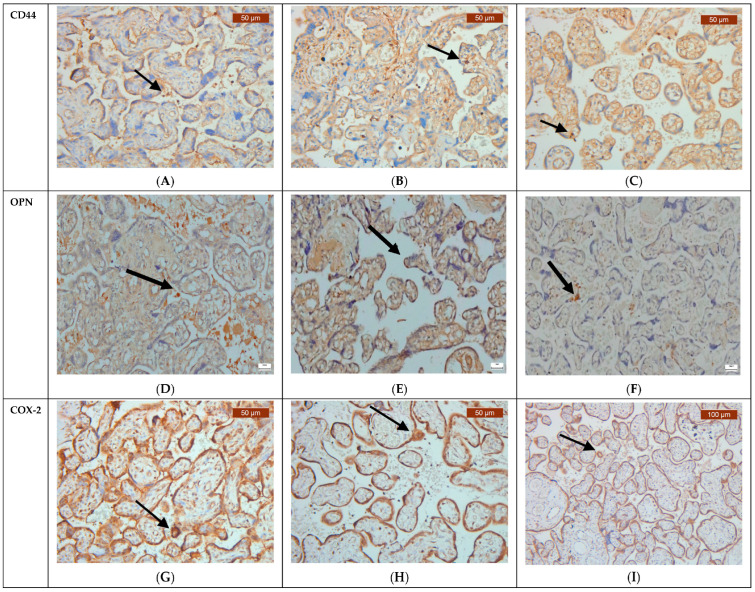
Representative immunohistochemical analysis of CD44, OPN, and anti-COX-2 in trophoblastic mononuclear cells. The arrows indicate positive staining. (**A**) COVID-19-positive pregnant women (×20). (**B**) COVID-19-vaccinated pregnant women (×20). (**C**) COVID-19-negative and unvaccinated pregnant women (×20). (**D**) COVID-19-positive pregnant women (×20). (**E**) COVID-19-vaccinated pregnant women (×20). (**F**) COVID-19-negative and unvaccinated pregnant women (×20). (**G**) COVID-19-posietive pregnant women (×20). (**H**) COVID-19-vaccinated pregnant women (×20). (**I**) COVID-19-negative and unvaccinated pregnant women (×20).

Strikingly, our analysis identified a more intensive CD44 expression on the surface of placental connective tissue stroma within healthy unvaccinated placental tissue (Figure 3C, Table 2). In placental tissues from COVID-positive pregnant women, we observed a more subtle decrease in CD44 expression (Figure 3B, Table 2). Comparatively, CD44 expression was the lowest in COVID-vaccinated placental tissues (Figure 3B, Table 2).

Furthermore, our analysis demonstrated an upregulation of OPN expression in the placental tissues of COVID-vaccinated women, evident in both the epithelial and stromal compartments (Figure 3E, Table 2). COVID-negative and unvaccinated women showed a slight upregulation of OPN expression in placental tissues (Figure 3F, Table 2). In contrast, the OPN expression in COVID-positive placentas was the lowest (Figure 3D, Table 2). Surprisingly, both healthy and COVID-positive cases registered similar percentages of OPN within the stromal and epithelial compartments of the trophoblast, suggesting a relatively stable, non-inflammatory state. However, as reflected in the table above, a significant proportion of the cases were OPN-negative, particularly among the COVID-positive and healthy unvaccinated groups. The most notable difference was observed in the vaccinated group, where the number of OPN-positive cases was double that of the OPN-negative cases (Table 2).

In COVID-negative and unvaccinated placentas, we detected an increased expression of anti-COX-2 in trophoblasts, decidua cells, and infiltrating immune cells. Following COVID infection, anti-COX-2 expression was modestly elevated compared to its low expression levels in vaccinated placentas, with no negative cases of anti-COX-2 in healthy and vaccinated pregnant women (Figure 3G–I, Table 2).

**Figure 4:**
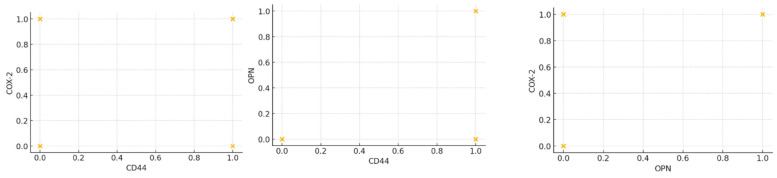
Scatterplots representing the correlations between our three biomarkers.

The correlations between the biomarkers were relatively low, indicating a weak linear relationship: 0.121 correlation between CD44 and OPN; 0.200 correlation between CD44 and COX-2; and 0.100 correlation between OPN and COX-2. The Cohen’s power analysis computed 87 participants per group to detect a medium effect size with a significance level of 0.05 and a power of 0.8. We used a simplified logistic regression model using only the main effects the groups and CD44, due to limited data that made it difficult to estimate the effects with accuracy. The results indicated that women from the vaccinated group were significantly more likely to have a positive OPN expression compared to those from the COVID-positive group, with no significant differences in OPN positivity between the control group and the COVID-negative group, and without any significant impact of CD44 positivity on the likelihood of OPN being positive. Fisher’s exact test results for each biomarker across our group comparisons reinforced our results, indicating that COVID vaccination is associated with a higher expression of CD44 and OPN, while COX-2 expression remains unaffected (Figure 4).

## 4. Discussion

A wide range of placental histopathologic abnormalities secondary to COVID infection during pregnancy have been reported in the literature, with central findings represented by vascular malperfusion and villitis. The fact that these placental alterations are present regardless of the symptomatic or asymptomatic status of the pregnant women suggests a direct viral causative effect. The presence of virions in the placental villis further supports this. A severe systemic inflammatory response and hypercoagulable state, with widespread microthrombi, induced by COVID infection, are certainties [1,4,6,15]. Garg et al., Debelenko et al., and Chen et al. described decidual arteriopathy, with utero-placental insufficiency and consecutive hypoxia, accompanied by fibrinoid necrosis, increased syncytial knots, and inflammatory villitis processes [5,16,17]. Information about placental injury in COVID-asymptomatic women is scarce, so we investigated whether this viral infection has a significant impact on cluster of differentiation 44 (CD44), osteopontin (OPN), and cyclooxygenase-2 (COX-2) placental inflammatory markers. We investigated the expression of these three key molecules in a placental study to clarify some of the ongoing debates surrounding previous epidemics.

Often, secondary tissue hypoxia, together with widespread anemia in pregnancy in our region, significantly decrease the oxygen transport capacity [18]. Although significant therapeutic efforts are devoted to preventing and correcting anemia in pregnancy, a high proportion of low hemoglobin levels in our entire cohort of pregnant women, regardless of their infectious or vaccinated status, was still observed. Up-to-date evidence suggests that COVID vaccination has a low impact on the placenta, without significant differences compared to healthy subjects [19].

Routine blood analysis is also impacted by this disease. The most encountered alteration in COVID infection is lymphopenia with elevated neutrophil count levels [20]. Although frequently described in the literature, our study did not detect such changes. This might have been due to the asymptomatic state of our patients, the small number of cases, or unknown region particularities.

Despite the fact that the literature has often described a multitude of unfavorable neonatal outcomes, our study only identified two: a lower birth weight and a lower gestational age at birth [3,20]. This was also reflected in the study by Maranto et al., 2023, which noted a higher prematurity rate in COVID-positive women, especially in symptomatic cases, with higher rates of delivery through cesarean sections in these cases [21], as in our research. Icognito et al. conducted a study that revealed variations in maternal–fetal severity of outcomes based on the COVID infection subtype. The findings highlighted the significant impact of early subtypes, like Delta, in contrast to later subtypes, such as Alpha and Omicron, with milder maternal–fetal impacts [22]. Maranto et al. underlined the importance of COVID vaccination in this reluctant but very vulnerable obstetric population, with the purpose of enhancing maternal–fetal outcomes [23]. Another aspect of interest to researchers is the fact that cesarean section is associated in some studies with an increased rate of neonatal secondary infection [24]. This was not observed in our results. Despite the fact that all of our neonates were accommodated with their mothers, and all of them were exclusively breastfed, none became infected. However, despite positive short-term fetal outcomes, we cannot exclude the possibility of long-term consequences arising from this disease [25].

The interaction between CD44 and HA is important in angiogenesis, particularly in the differentiation of endothelial cells, but also in their proliferation and migration [8,26]. This seems to play a central role, especially in the first trimester of pregnancy, with CD44 having a high degree of expression in almost every placental structure (decidua, syncytial knots and bridges, fibroblast and muscle cells, and mucosal connective tissue) [8,27]. Our results reflected an increased CD44 expression in COVID-negative unvaccinated pregnancy placentas, suggesting that in these cases placentation evolves in a natural physiological manner. The alterations in placental architecture and angiogenesis, consequent to COVID infection and vaccination, are reflected by a more discrete presence of CD44 in the epithelial and stromal compartments.

Another interesting aspect is the fact that placental first trimester macrophages, the Hofbauer cells, with less accurate detected functions, secrete a number of placental angiogenesis and remodeling molecules, such as OPN. OPN seems to be involved in implantation and placentation, favoring the angiogenesis process [11,28]. Our results showed that the majority of our pregnant women, regardless of group, had a negative expression of OPN in their placentas. The rates of positive cases were similar between COVID-positive and COVID-negative cases. This might have been secondary to our case enrolment process, as we did not separately analyze cases with COVID in each trimester of pregnancy, and perhaps more pronounced OPN positivity results from an early viral infection, with altered placentation and Hofbauer cell function. However, COVID vaccinations administered during earlier gestation led to subtle alterations in the proportion of OPN-positive cells, with the number of positive cells nearly double the negative cells.

Increased COX-2 expression in tissues is related to an inflammatory response. This molecule has a markedly low expression in normal tissue but dramatically increases in some conditions, related to an inflammatory state [29,30,31,32,33]. COX-2 is a central player in the human decidualization process, as a modulator of mitogenesis, differentiation, and angiogenesis [33,34]. A deficit is linked to unfavorable pregnancy outcomes [30,31,32,35]. Our findings clearly underlined high COX-2 positivity in normal unaffected unvaccinated pregnancies compared to COVID-positive or vaccinated cases. The downregulation of COX-2 in vaccinated cases, although subtler, might have been secondary to reduced inflammation and immunomodulation due to the vaccine.

Zika infection shares many similarities with COVID but more seriously affects fetal development due to its high tropism in trophoblastic cells, causing inflammation and metabolic impairment with mitochondrial dysfunction. COVID infection has modestly negative pregnancy outcomes, with severity varying depending on the viral subtype and mother’s symptoms [18,36]. Despite this, research is still needed to fully understand the disease and better prepare for possible future pandemics. The significant differences between the groups in the CD44 and OPN status suggested that vaccination may enhance certain immune pathways, potentially offering greater protection or a more vigilant immune state compared to natural infection or the unvaccinated state. This has implications for understanding the long-term benefits of vaccination in terms of immune system activation.

Our study had limitations due to the limited number of cases and the lack of a separate pregnancy trimester analysis. However, data from the literature remain scarce and we believe that our results are meaningful. Further research is essential to fully elucidate the implications of the expression of these markers and to confirm these findings.

## 5. Conclusions

The findings in this work reflected an alteration in the placental structure and consequent function due to altered expression of the three studied molecules. We detected high levels of CD44 and COX-2 expression in normal uninfected and unvaccinated pregnancies as a result of the normal process of placentation, with downregulation of these molecules in COVID-infected women and more subtle changes in vaccinated individuals. The OPN levels remained similar, potentially due to late pregnancy COVID infection with less affected placentation. This underlines the need for closer obstetric monitoring due to a high risk of unfavorable maternal and fetal outcomes.

## Figures and Tables

**Table 1:** Clinical and demographic characteristics of the pregnant women.

Clinical and Demographic Characteristics of the Women		No.	Mean	St. Dev.	Percentile 25	Median	Percentile 75	Min	Max	Kruskal–Wallis *p*-Value
Maternal age (years)	Negative and unvaccinated	70	28	4	27	29	31	19	34	<0.001
	Positive	65	31	5	28	33	34	18	39	
	Pfizer vaccinated	35	30	5	27	30	33	18	39	
Gestational age at delivery (weeks)	Negative and unvaccinated	70	40	1	38	40	41	37	41	<0.001
	Positive	65	38	3	37	38	39	28	40	
	Pfizer vaccinated	35	38	3	37	39	40	29	41	
Fetal weight (grams)	Negative and unvaccinated	70	3507	354	3260	3495	3670	2800	4490	0.01
	Positive	65	3237	536	2920	3300	3600	1980	4540	
	Pfizer vaccinated	35	3291	522	2920	3390	3650	1990	4570	
Apgar score at 1 min (point)	Negative and unvaccinated	70	9	1	8	9	9	8	9	0.67
	Positive	65	8	1	8	9	9	4	9	
	Pfizer vaccinated	35	8	1	8	9	9	4	10	
Hemoglobin antepartum (milligrams/deciliter)	Negative and unvaccinated	70	12.3	1.1	11.8	12.1	13.6	10.0	13.7	0.76
	Positive	65	12.2	0.8	11.9	12.3	12.5	9.7	13.4	
	Pfizer vaccinated	35	12.3	0.9	11.9	12.3	13.0	9.8	14.3	
Hematocrit antepartum (%)	Negative and unvaccinated	70	36.3	3.4	35.0	37.0	38.9	28.1	40.0	0.578
	Positive	65	36.4	2.6	35.3	36.0	38.0	29.3	41.0	
	Pfizer vaccinated	35	36.8	2.9	35.3	36.9	38.8	28.1	42.1	
Hemoglobin postpartum (milligrams/deciliter)	Negative and unvaccinated	70	10.9	0.8	10.4	10.9	11.4	9.0	13.0	0.61
	Positive	65	11.0	0.9	10.4	11.0	11.6	8.9	12.8	
	Pfizer vaccinated	35	11.1	0.8	10.5	11.0	11.6	9.7	12.9	
Hematocrite postpartum (%)	Negative and unvaccinated	70	31.8	2.9	29.0	31.1	34.3	27.8	37.0	0.52
	Positive	65	32.3	2.8	30.4	32.3	33.6	27.7	38.5	
	Pfizer vaccinated	35	32.4	2.8	29.9	32.5	34.3	27.5	37.5	
Leucocyte value (10^3^/L)	Negative and unvaccinated	70	12,293	2272	10,350	11,485	14,600	9900	16,500	<0.001
	Positive	65	10,186	2590	8280	10,120	12,100	5390	16,400	
	Pfizer vaccinated	35	10,936	2922	8320	10,350	12,280	5390	16,500	
Platelet value (10^6^/L)	Negative and unvaccinated	70	220,500	78,240	149,000	217,500	278,000	122,000	355,000	0.76
	Positive	65	218,523	63,791	156,000	211,000	260,000	132,000	355,000	
	Pfizer vaccinated	35	227,543	71,222	156,000	218,000	278,000	135,000	360,000	
CRP (C-reactive protein) value (milligrams/deciliter)	Negative and unvaccinated	70	3.12	1.52	2.00	2.75	4.10	0.90	10.00	0.89
	Positive	65	3.09	1.30	1.90	3.00	4.80	1.07	4.90	
	Pfizer vaccinated	35	3.18	1.20	2.30	2.80	4.10	1.00	4.90	

**Table 2:** CD44, OPN, and anti-COX-2 placental expression in our three groups of pregnant women.

			CD44 (Cluster of Differentiation 44)		*p*-Value	OPN (Osteopontin)		*p*-Value	COX-2 (Cyclooxygenase-2)		*p*-Value	
			Negative	Positive		Negative	Positive		Negative	Positive		Total
Group	COVID-positive	Count	23	42	<0.001 * Positive vs. vaccinated	53	12	<0.001 * Positive vs. vaccinated	4	61	0.295 F Positive vs. vaccinated	65
		% within lot	35.4%	64.6%		81.5%	18.5%		6.2%	93.8%		100.0%
	Pfeizer vaccinated	Count	0	35	0.004 F Vaccinated vs. negative	11	24	<0.001 * Vaccinated vs. negative	0	35	All positive Vaccinated vs. negative	35
		% within lot	0.0%	100.0%		31.4%	68.6%		0.0%	100.0%		100.0%
	Negative and unvaccinated	Count	14	56	0.045 * Positive vs. negative	56	14	0.82 * Positive vs. negative	0	70	0.051 F Positive vs. negative	70
		% within lot	20.0%	80.0%		80.0%	20.0%		0.0%	100.0%		100.0%
Total		Count	37	133		120	50		4	166		170
		% within lot	21.8%	78.2%		70.6%	29.4%		2.4%	97.6%		100.0%

F—Fisher’s exact test. *—Chi-square test.

## Data Availability

The data used to support the findings of this study are available upon request to the corresponding author.
